# Prevalence and associated characteristics of recurrent non-specific low back pain in Zimbabwean adolescents: a cross-sectional study

**DOI:** 10.1186/1471-2474-15-381

**Published:** 2014-11-19

**Authors:** Matthew Chiwaridzo, Nirmala Naidoo

**Affiliations:** Department of Rehabilitation, University of Zimbabwe, College of Health Sciences, Harare, Zimbabwe; Division of Physiotherapy, Department of Health and Rehabilitation Sciences, University of Cape Town, Faculty of Health Sciences, Cape Town, South Africa

**Keywords:** Adolescents, Lifetime prevalence, Non-specific low back pain, Point prevalence, Recurrent non-specific low back pain

## Abstract

**Background:**

Until recently, non-specific low back pain (NSLBP) in adolescents was considered a rare phenomenon unlike in adults. The last two decades has shown an increasing amount of research highlighting the prevalence in this age group. Recent studies estimate lifetime prevalence at 7%-80%, point prevalence at 10%-15%, and prevalence of recurrent NSLBP at 13%-36%. In Zimbabwe, there is dearth of literature on the magnitude of the problem in adolescents. Therefore, the aims of the study were to determine the prevalence (lifetime, point, recurrent) and the nature of recurrent NSLBP reported by adolescents in secondary schools.

**Methods:**

A cross-sectional study was conducted using a questionnaire. A cluster sample of 544 adolescents (age 13–19 years) randomly derived from government schools participated in the study. Lifetime prevalence, point prevalence and prevalence of recurrent NSLBP were presented as percentages of the total population. Exact 95% confidence intervals were given. Chi-square test was used to evaluate the effect of gender and age on prevalence.

**Results:**

The students’ response rate was 97.8%. The lifetime prevalence was 42.9% [95% confidence interval = 40.8-44.6] with no significant difference between sexes [*χ*^*2*^ (1) =0.006*, p* = 0.94]. However, NSLBP peaked earlier in female students (13.9 years) than in male students (15 years) [*t* (226) = 4.21, p < 0.001]. About 10% of the adolescents reported having an episode of NSLBP on the day of the survey. However, female students (14.2%) were more affected on the day [*χ*^*2*^ (1) *=* 11.2*, p <* 0.001]. Twenty-nine percent of the adolescents experienced recurrent NSLBP with 78% experiencing at least three episodes in the last 12 months. On average, recurrent NSLBP reported was mild in intensity (4.8 ± 1.9) on the visual analogue scale (VAS) and short in duration. Recurrent NSLBP was associated with sciatica in 20.9% of adolescents.

**Conclusions:**

NSLBP is a common occurrence among Zimbabwean adolescents in secondary schools. It increases with chronological age and is recurrent in the minority of adolescents. Although much of the symptomatology may be considered benign, the existence of recurrent NSLBP in adolescents before their work-life begins should be a concern to health professionals, teachers and parents.

**Electronic supplementary material:**

The online version of this article (doi:10.1186/1471-2474-15-381) contains supplementary material, which is available to authorized users.

## Background

Non-specific low back pain (NSLBP) has been widely described as pain or discomfort that is localised below the costal margin and above the inferior gluteal folds, with or without leg pain, but not attributable to a known or specific pathology [1]. Globally, it has been identified as an important public health problem among adults with an estimated lifetime prevalence of over 60% associated with adverse consequences [2–4]. Over the last two decades, research highlighting the prevalence of NSLBP in the young population has increased as evidenced by substantial literature published on the matter. The evidence portrays a different perspective on the age of onset of NSLBP in the general population [5].

The first episodes of NSLBP could be experienced as early as nine years of age, and continue into adulthood [5, 6]. However, the absolute lifetime prevalence estimates varied between studies [7–10]. This applied even for studies sharing similar definition of lifetime prevalence and similar methodological design. Cross-sectionally, the lifetime prevalence has been reported to be 61% in Spain, 65% in Norway, 40% in the United Kingdom and 34.5% in the United States of America [11–14]. In low-income countries, the lifetime prevalence has been similarly reported: 58% in South Africa, 57.8% in Kuwait and 25% in Nigeria [15–17]. Lifetime prevalence has been described to indicate the proportion of people that experience an episode at one point in life [13]. However, about 10%-15% of adolescents report NSLBP at a specific point in time [3, 13].

To our knowledge, the dearth of literature on the condition in Zimbabwe is a significant shortcoming. This could be attributed to entrenched beliefs that adolescent NSLBP is an inevitable experience of growing. In contrast, recent evidence highlight that 13%-36% of adolescents will suffer recurrent NSLBP with negative impact on health and function [13, 15, 18–20]. Lack of standard definition on recurrent NSLBP partly explains this, with majority of studies relying on random or pragmatic definitions [13, 21]. Nevertheless, recurrent NSLBP has been characterised by variable pain intensity and short duration episodes [13, 16, 20, 22–24]. In Zimbabwe, the exact nature of recurrent NSLBP in adolescents is unknown. Against that background, the primary specific objective of the study was to determine the lifetime prevalence, point prevalence and prevalence of recurrent NSLBP in adolescents in schools. The secondary objective was to determine the nature of recurrent NSLBP with regards to frequency, intensity, duration of episodes, presence or absence of sciatica and the health-seeking behaviour of adolescents with the condition.

## Methods

### Study design

The study used a cross-sectional design and was conducted in government secondary schools based in Harare, Zimbabwe. The government-administered secondary schools are classified into two categories (S_1_ and S_2_) based on location. The S_1_ schools are located in the low density areas where people of high socio-economic status live [25]. The S_2_ schools are located in the high density suburbs where people of lowest socio-economic status reside [25]. At the time of the study, a total of 71 458 adolescents were registered in government secondary schools.

As no study has been conducted in Zimbabwe on adolescent recurrent NSLBP, Epi Info version 7.1.1.0 Statcalc package for population surveys was used to calculate the minimum sample size. The following parameters were considered: target population (71 458), estimated prevalence of recurrent NSLBP in school-children found regionally (13.5%) [26], precision effect 3%, design effect (1%) and 95% confidence interval. The sample size was calculated to be 495. However, the number was adjusted upwards in anticipation of attrition from school absenteeism and possible refusals. The final sample size was 620 students.

A two-stage cluster sampling method was used to select schools and study participants. There are 55 relevant government-administered secondary schools in Harare, Zimbabwe. There are 17 secondary schools in the S_1_ and 38 secondary schools in the S_2_ category. In the first-stage of sampling, a list of all government secondary schools was constructed in clusters of two categories, S_1_ and S_2_. Each school in the two clusters was assigned a numerical number. Based on a strategy that considered proportionality between the S_1_ and S_2_ schools, one secondary school was randomly selected from the S_1_ category and two from S_2_ cluster box.

In the second stage of sampling, each school selected was asked to provide a list of all classes in each form. Six classes were randomly selected where all the school-children in the relevant classes were then eligible to participate in the study. Inclusion criteria considered adolescents registered full-time in the participating schools. The World Health Organisation (WHO) definition of adolescents was adopted as people ranging between 10 and 19 years [16]. In addition, school-children eligibility entailed obtaining parental/legal guardians’ permission and submission of a completed medical health questionnaire. However, participants were excluded if they had parental reports of spinal pathologies, orthopaedic conditions, a previous history of trauma to the back, any central or peripheral nervous problem affecting muscle quality in the extremities and trunk. In addition, they were excluded if they had overt or covert physical deformities such as leg length discrepancy, scoliosis and lordosis [27].

### Low back pain questionnaire

As there was no Zimbabwean questionnaire on adolescent recurrent NSLBP, questions asked in the questionnaire were derived from previously validated instruments used in literature [10, 16, 27–29]. The choice of a questionnaire as a survey tool was based on the fact that pain is a highly subjective phenomenon best evaluated by self-report [29–31].

The first section of the questionnaire gathered information on the primary outcome measures of lifetime prevalence, point prevalence and the prevalence of recurrent NSLBP. For lifetime prevalence, respondents were specifically asked the following question “Have you ever experienced pain or discomfort in the lower part of your back which lasted for one day (24 hours) or longer in your life, not associated with menstruation in females?” To assist the respondents in understanding the anatomical region of the lower back, a mannequin was used with an arrow pointing to a posterior view of the lumber region. Subsequently, respondents were asked to provide the age when they first experienced an episode of low back pain in order to determine the mean age of onset of the condition.

For point prevalence, respondents were specifically asked the following question “Do you have pain in the lower part of your back now?” The operational definition of recurrent NSLBP adopted was “pain which occurred at least two times over the past year with each episode of lasting at least 24 hours, with pain intensity of greater than two on VAS with at least a 30-day pain free period between the episodes” [21]. Recurrent NSLBP was specifically asked regarding the last 12 months. In addition, to understand the nature and the character of the recurrent NSLBP experienced by adolescents, the researcher sought information on the frequency, intensity and the duration of the recurrent episodes. Pain intensity was evaluated based on the VAS from 0 (no pain) to 10 (maximum pain). The health-seeking behaviour for adolescents with recurrent NSLBP was also ascertained by the following question “Have you ever sought treatment for non-specific recurrent low back pain or information from any health-care professional regarding the pain that you frequently feel? This behaviour described seeking formal or informal health care services.

### Medical health questionnaire

The medical health questionnaire was used to determine the medical history of school-children as reported by parents or legal guardians. The questionnaire was adapted and modified to suit the design of the research study from a study conducted by Fanucchi et al. [27]. The questionnaire provided the criteria for exclusion from the study. Students were excluded if they had spinal pathologies, or deformities such as scoliosis, spinal tumours, spinal trauma, and orthopaedic conditions such as fractures of the pelvis and lower limbs, leg length discrepancy and any neurological conditions which alter muscle tone of the lower limbs and the spine. These conditions fell out of the scope of the study.

### Instrument development

Prior to use, the low back pain study questionnaire was subjected to content validity testing and one-week test-retest reliability test. Five lecturers from the Department of Nursing, Physiotherapy and Community Medicine at the University of Zimbabwe were used as content experts. They were chosen on the basis of experience in epidemiological research studies and on preferential interest in musculoskeletal research. Subsequently, the questionnaire was administered to a sample of 40 students twice. During the initial test, students were not told that they would be re-tested again after one week. For the primary outcome measures of NSLBP, moderate to substantial kappa coefficients (0.48-0.72) were observed (Table 1). These results were consistent with findings from other studies [29, 32]

**Table 1:** **Kappa coefficients of the items in the low back pain questionnaire for adolescents (n = 40)**

Question	Kappa value	Comment
Q1: Lifetime prevalence	0.72	Substantial
Q3: Prevalence of Recurrent NSLBP	0.51	Moderate
Q4: LBP Frequency	0.73	Substantial
Q5: LBP Duration	0.96	Perfect
Q7: Sciatica	0.32	Fair
Q8: Period prevalence	0.48	Moderate
Q10: Medical Treatment	0.56	Moderate
Q12: School Absenteeism	0.88	Perfect
Q13: School-bag	1	Perfect
Q14: School-bag weight perception	0.74	Substantial
Q15: Duration of carrying school-bag	0.86	Perfect
Q16: Method of carrying school-bag	0.83	Perfect
Q17: Sports participation	1	Perfect
Q19: Sports duration	1	Perfect
Q20: Sedentary time	0.60	Perfect
Q21: Smoking status	0.83	Perfect

### Procedure

The study was approved by the Human Research Ethics Committee (HREC) from the University of Cape Town **[ref: 189/2012]**, Medical Research Council of Zimbabwe (MRCZ) **[ref: MRCZ/B/356]**, Ministry of Education, Sports, Arts and Culture **[ref: C/426/3]** and Harare Provincial Education Director **[ref: G/42/1]**. School-children as well as their parents/legal guardians gave assent and consent respectively to participate in the study. The fieldwork for the first phase of the study was conducted during the second academic school term. The fieldwork was subdivided into three distinct stages: preparatory stage, intermediate stage, and questionnaire administration.

### Preparatory stage

After the schools had agreed to participate, the researcher (MC) visited the schools to establish background information. The researcher utilised this opportunity to address school authorities on the nature and procedural issues of the research project and to agree on specific dates for data collection. In addition, the researcher utilised the opportunity to identify the participating classes and students in each of the participating secondary schools for the main study.

### Intermediate stage

On an agreed date, the researcher attended the selected schools to address the participating students. Verbal information was given explaining the rationale, procedure, risks and benefits of the research project to the students. At that stage, students who verbally agreed to participate were given a letter with standard information regarding the study, and consent forms for parents/legal guardians. In addition, students were given the medical health questionnaire to be completed by parents/legal guardian at home. The students were given seven days to return the parental documents to the class teacher in a sealed envelope. During this period, reminders were sent to the respective class teachers using cell-phone text messages and voice calls to ensure prompt return of the documents by students. The researcher collected returned parental documents from the class teachers intermittently to the end of the seven days.

### Questionnaire administration

On agreed dates with the school authorities, the researcher visited the three participating schools consecutively. Because each school had six participating classes, a maximum of three days of data collection were spent at each school. This was done to minimise disruptions of scheduled lessons. Eligible students were given an information sheet to read then sign an assent form. The questionnaires were completed in the classrooms during school hours in the presence of the researcher and the class teacher.

### Statistical analyses

Raw data from the questionnaires was entered into Microsoft Excel before being imported to STATISTICA version 11 for analysis. Questionnaires with missing data were not analysed. Descriptive statistics were used to describe baseline demographic characteristics of the participants, including means with standard deviations for continuous data and frequencies for categorical data. The primary outcome measures of lifetime prevalence, point prevalence and prevalence of recurrent NSLBP were expressed as percentage of the total population. Exact 95% confidence intervals (CI) were given. Chi-square test was used to evaluate the effect of gender and age on NSLBP prevalence. The severity of recurrent NSLBP was evaluated using VAS and the independent *t*-test was used to assess the difference between males and females. Pain intensity was dichotomised as mild pain (1–4 score on VAS) and severe pain (5–10 score on VAS) [16].

## Results

**Figure 1:**
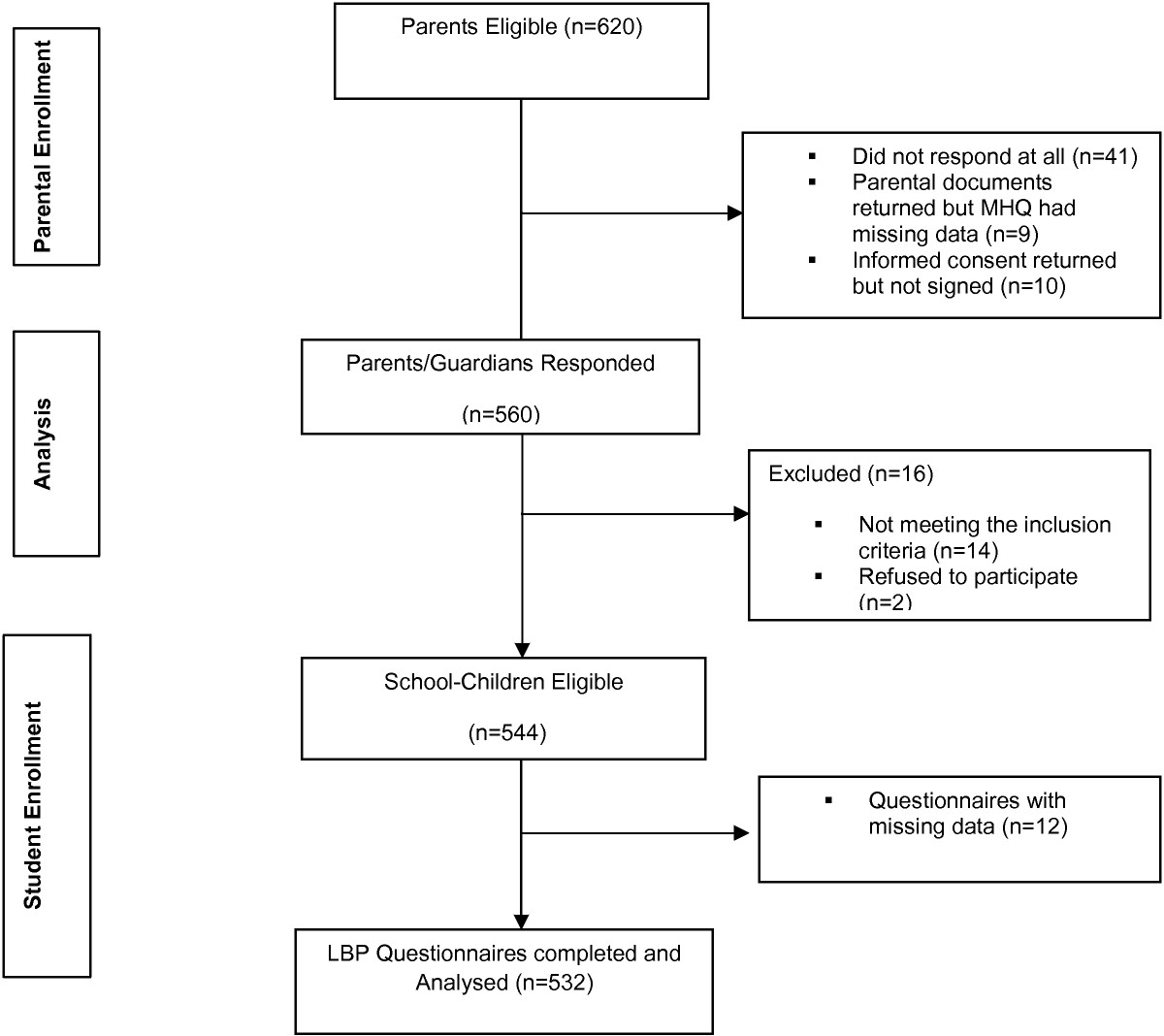
**Flow chart showing participants from parental enrolment to adolescents’ enrolment.**

Figure 1 outlines the questionnaire distribution and return. Of the 620 parents eligible, 560 parents returned the parental documents (Informed consent and the medical health questionnaire) allowing for child participation. Analysis of the parental documents ensured that 14 school-children were excluded for failing to meet the inclusion criteria. Two school-children refused to participate. However, of the 544 school-children eligible for the study, 532 (97.8%) completed the survey instrument.

### Baseline characteristics for participants

Table 2 depicts the demographic characteristics of the study participants. The mean age of the sample was 16 ± 1.72 years (range 13–19 years). Of the total school-children, 53.8% (n = 286) were female students. As indicated by the independent *t*-test, there was a significant difference in the mean age of participants between male students (16.2 ± 1.79) and female students (15.8 ± 1.65) [*t* (530) = 2.34, p = 0.02].

**Table 2:** **Baseline characteristics for the study participants (N = 532)**

Characteristic	Total	%	Cumulative %
Females	246	46.2	
Males	286	53.8	
Age (years)			
13	29	5.4	5.4
14	98	18.4	23.8
15	101	19.0	42.8
16	96	18.0	60.8
17	90	16.9	77.7
18	67	12.6	90.2
19	51	9.6	100.0
Form (Years of education)			
1 (8)	74	13.9	13.9
2 (9)	116	21.8	35.7
3 (10)	106	19.9	55.6
4 (11)	100	18.8	74.4
5 (12)	77	14.4	88.8
6 (13)	59	11.2	100

### Prevalence rates of non-specific low back pain

**Figure 2:**
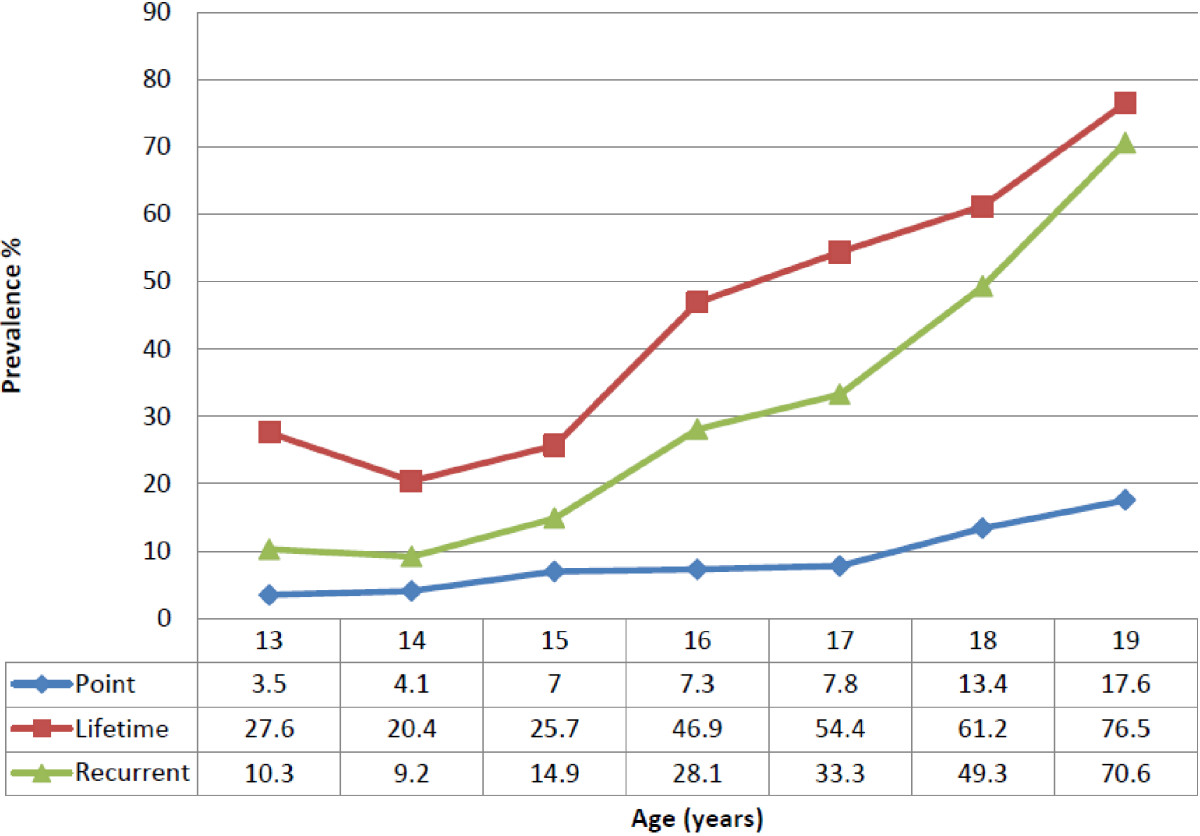
**A summary of lifetime (n = 532), point (n = 532) and recurrent (n = 153) prevalence figures across all age ranges.**

**Figure 3:**
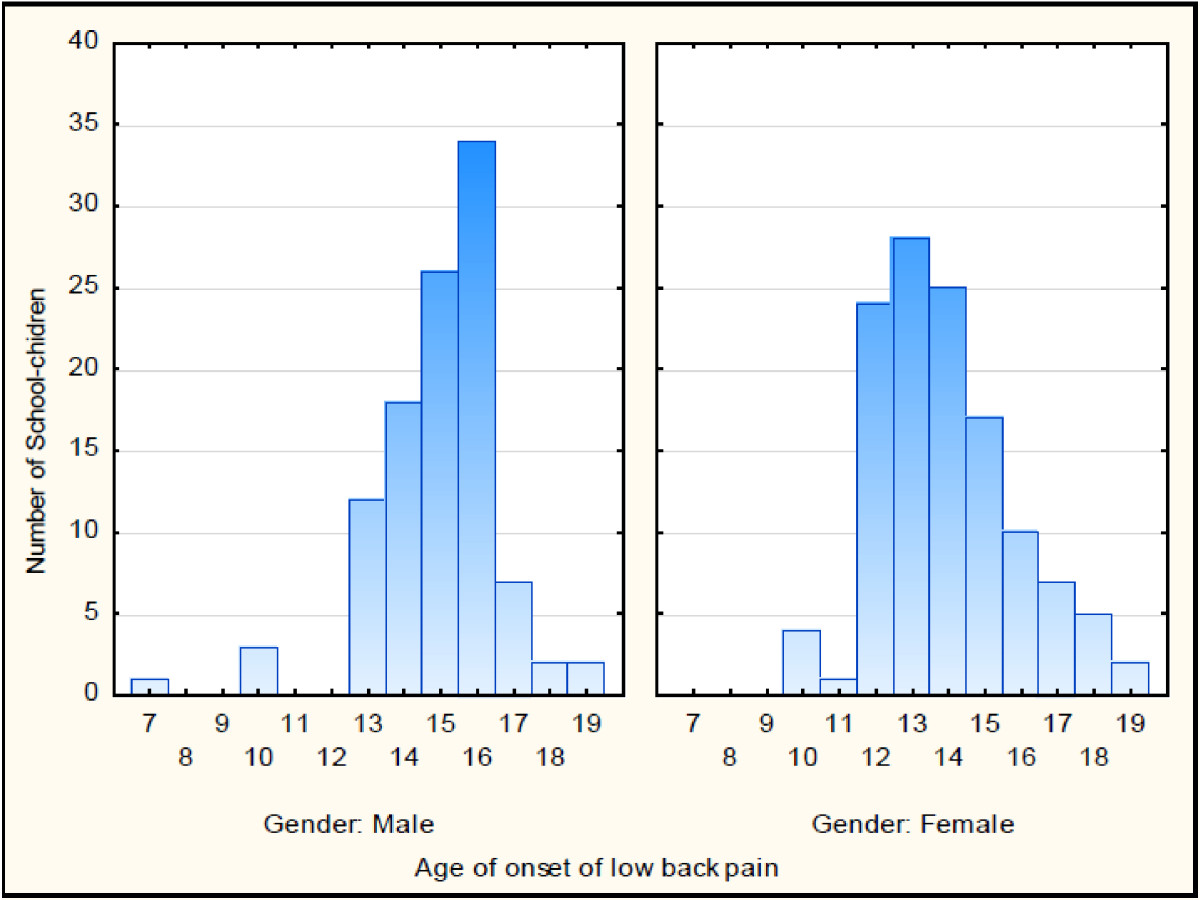
**The difference in the age of onset for lifetime non-specific low back pain between males and females (n = 532).**

The lifetime prevalence was 42.9% (Table 3). There was no significant difference in the lifetime prevalence between male students (42.7%) and female students (43.0%) as indicated by the Chi-square test [*X*^2^(1) = 0.006, p = 0.94]. There was a significant trend towards increase in the proportion of school-children with NSLBP as age increased despite some inconsistencies at some ages [*X*^2^ for linear trend = 73.3, p < 0.001] (Figure 2). The mean age of onset for NSLBP in the school-children was 14.4 ± 1.90 years. There were different peaks of onset between the sexes. Figure 3 shows that NSLBP peaked earlier in female students. The mean age at onset was 13.9 ± 1.91 years for female students compared to 15.0 ± 1.75 years for male students [*t*(226) = 4.21, p < 0.001].

**Table 3:** **Prevalence results for non-specific low back pain in adolescents**

Age (yrs)	Lifetime prevalence (n = 532)		Point prevalence (n = 532)		Recurrent prevalence (n = 153)	
	Males (%)	Females (%)	Males (%)	Females (%)	Males (%)	Females (%)
13.0-13.9	26.7	28.6	2.0	5.0	6.7	14.3
14.0-14.9	17.6	21.9	2.9	4.7	11.8	7.8
15.0-15.9	20.0	31.4	4.0	9.8	16.0	13.7
16.0-16.9	42.9	50.0	2.4	11.1	21.4	33.3
17.0-17.9	54.3	54.5	5.7	9.1	28.6	36.4
18.0-18.9	61.5	60.7	5.1	25	51.3	46.4
19.0-19.9	77.4	75.0	6.5	35	67.7	75.0
Average	42.7	43.0	4.1	14.2	29.7	28.0
95% CI	40.8-44.6	41.4-44.6	3.9-4.2	13.2-15.2	27.8-31.6	26.0-30.0

Point prevalence indicated that 9.2% of the school-children had NSLBP on the day of questionnaire adminstration. Female students were affected significantly compared to male students [*X*^*2*^ (1) = 11.2, p < 0.001]. Overall, there was a significant trend towards increase in the proportion of school-children with point NSLBP as age incresaed [*X*^*2*^_trend_ = 29.8, p < 0.001] (Figure 2). The mean intensity of point NSLBP was 3.5 ± 1.64 on the VAS. An independent *t-* test showed no significant difference between male students (3.08 ± 1.12) and female students (3.68 ± 1.76) in the intenstity [*t*(51) = -1.15, p = 0.26]. In adittion, as indicated by the one-way analysis of variance (ANOVA) test, there were no significant differences in the mean intensity of point NSLBP across the age categories [F (6,46) = 1.69, p = 0.14].

The prevalence of recurrent NSLBP was 28.8%. Male students were more affected (29.7%) compared to female students (28.0%), although this was not statistically significant [X^2^ (1) = 0.19, p = 0.67]. The prevalence of NSLBP increased with age despite a slight drop for the 14 year olds (Figure 2).

### Characteristics of recurrent non-specific low back pain

Table 4 presents data on the characteristics of recurrent NSLBP. The majority of school-children (n = 82, 53.6%) experienced more than three episodes in 12 months. However, episodes of recurrent NSLBP were reported to last for less than a week in the majority, with both male and female students equally affected. Of the 153 school-children with recurrent NSLBP, 26.8% (n = 41) reported having sought medical treatment for the symptoms. Health-seeking behaviour was not gender-related. However, it was significantly related to the report of sciatica [*χ*^*2*^ (1) =47.9*, p* < 0.001]. Sciatica was reported in 20.9% (n = 32) of school-children with recurrent NSLBP. The mean intensity of recurrent NSLBP reported was 4.8 ± 1.94 on the VAS. Female students reported to experience intense episodes of recurrent NSLBP [t (151) = -5.66*, p <* 0.001].

**Table 4:** **Characteristics of recurrent non-specific low back pain (n = 153)**

Characteristic	Responses	Males (%)	Females (%)	*Χ* ^*2*^	*p*
	Twice	17.8	25.0	1.78	0.41
Frequency	Thrice	28.8	21.3		
	> Thrice	53.4	53.8		
	1-7 days	84.9	86.3	1.26	0.74
	8-14 days	11.0	10.0		
Duration	15-21 days	4.1	2.5		
	22-28 days	0	1.3		
	≥ 1 month	0	0		
	Yes	23.3	18.8	0.48	0.49
Sciatica	No	76.7	81.2		
	Yes	27.4	26.4	0.03	0.87
Treatment	No	72.6	73.6		

Health-seeking behaviour was significantly associated with pain intensity [*X*^*2*^ (1) = 6.55, p = 0.01]. Moreover, there was a significant difference in the mean intensity of recurrent NSLBP reported by adolescents who sought treatment compared to those who did not [*t* (151) = -5.03*, p* < 0.001].

## Discussion

This was a novel study in Zimbabwe to ascertain the prevalence of recurrent NSLBP and determine its nature in adolescents. The response rate from parents and adolescents was satisfactory. Bias due to non-participation could not have influenced the observed results. Interplay of factors could have contributed to the high response rates. The self-administration of the study questionnaires to adolescents in schools could have had a positive impact. In addition, parents were informed of the study having had formal approval from the school principals. This could have encouraged them to participate in a school-based project that evaluated the health of their school-child.

### Lifetime prevalence

The lifetime prevalence rate observed in this study falls within the range (7%-80%) reported in systematic reviews of adolescent NSLBP studies [3, 8, 9, 23]. Variations in the prevalence figures have been attributed to methodological or population differences [9]. Using a relatively small sample size, a lifetime prevalence of 57.8% was reported in 400 school-children aged between 10 and 18 years in Kuwait [15]. These findings were consistent with results obtained in South Africa using a relatively larger sample of 1 123 adolescents between the ages of 13 and 18 years [17]. In contrast, a large cross-sectional study conducted among 43 630 school-children aged between 9 and 15 years, found a relatively lower lifetime prevalence of 32.1% in Japan [33]. However, the study could be criticised on the grounds that the authors left the judgment of NSLBP to the respondents. In addition, the sample was relatively younger compared to the present study sample. Nevertheless, the possibility that lifetime prevalence could have been under-reported in the present study cannot be over-ruled. This is because the screening question on lifetime prevalence had a kappa coefficient of 0.72 indicating substantial but not perfect agreement. This finding highlights the possible existence of recall bias secondary to a phenomenon described as memory decay [34]. It is possible that adolescents forget past lifetime episodes of NSLBP resulting in under-estimated prevalence figures.

The prevalence increased with advancing age despite inconsistencies at some ages. This has been reported in other cross-sectional studies [13, 16]. This indicates that NSLBP becomes increasingly common with age. These findings together with a well-known fact that adolescent NSLBP predicts adult NSLBP expose a significant future health-care problem in Zimbabwe [23]. In addition, NSLBP affects males and females equally during adolescence. This is consistent with other studies. A cross-sectional study conducted in Nigeria reported lifetime prevalence figures of 59.7% and 57.5% for male and female students respectively [χ^2^ = 1.49, p = 0.222] among 4 400 school-children aged between 10 and 19 years [16]. There is no explanation that has been postulated in the literature for this [16]. In contrast, the majority of studies indicate to a higher prevalence of NSLBP in females compared to males [11, 30, 35]. Other studies report a higher prevalence in males [17, 36]. Although the actual reasons for the higher prevalence of spinal pain among females are unknown, several authors have attributed this to menses and accelerated growth spurt [27, 37, 38]. On the other hand, Jordaan et al. [17] cited higher concentration of testosterone during pubertal growth in males for the higher prevalence.

### Recurrent non-specific low back pain

A small subset (28.8%) of adolescents reported having experienced recurrent NSLBP in the last 12 months. Both sexes were equally affected and the prevalence increased with chronological age. These findings provide support to recent epidemiological reports that NSLBP is recurrent in nature and confirms the indiscriminate nature of the condition [10, 13]. There is sparse data available regarding the prevalence of recurrent NSLBP in adolescents in the literature. The few studies available have reported different rates because of definitional issues. This makes comparison between studies difficult [13].

In Norway, recurrent NSLBP was reported to be 32% in adolescents with mean age of 14.7 years [18]. Apart from the small sample size used (n = 88), the operational definition of recurrent NSLBP considered pain experienced for more than seven days for over 12 months. There was no specific emphasis on the intensity and frequency of episodes that constituted a recurrence of NSLBP. In contrast, a cross-sectional study conducted in the United Kingdom among 500 school-children aged 10 and 16 years reported a lower prevalence of 13.1% [13]. Recurrent NSLBP was specified as pain experienced “regularly” without clarity on duration, intensity and frequency of episodes [13]. Similarly, a study conducted in Mozambique reported a recurrent prevalence of 13.5% in a study involving 204 school-children aged between 11 and 16 years [26].

The seemingly high prevalence of recurrent NSLBP in Zimbabwean adolescents could be attributed to several factors. Recurrent NSLBP may indeed be a problem of concern among Zimbabwean adolescents. Since the study had no monetary benefits, it is highly unlikely that the school-children exaggerated or reported a non-existent problem. However, due to the relatively small sample size used (n = 153) and the cross-sectional nature of the study, it is possible that the 12-month prevalence of recurrent NSLBP could have been overestimated through a concept of forward telescoping [34]. The reliability of the screening question for recurrent NSLBP showed moderate agreement (k = 0.51) between the test and re-test. In addition, the adopted definition of recurrent NSLBP has not been validated in adolescents.

### Characteristics of recurrent non-specific low back pain

A number of parameters were evaluated to assess the characteristics of recurrent NSLBP reported in Zimbabwean adolescents. This was done to understand the nature of recurrent NSLBP in adolescents with regard to frequency, intensity, and duration of episodes. However, a limited number of studies have specifically examined these aspects of recurrent NSLBP. In the present study, more than half (n = 82) of the school-children with recurrent NSLBP reported having experienced at least three episodes of NSLBP in 12 months. This strongly indicates that recurrent NSLBP is a common phenomenon and adolescents are vulnerable to many recurring episodes in a period of time; a finding shared by many authors [13, 23, 24]. In the majority of adolescents (85.6%), an episode of NSLBP was reported to last for a short period of time (less than seven days). Rarely was an episode reported to last for more than two weeks. This could reflect a favourable natural history of recurrent NSLBP in adolescents, a finding supported by Burton et al. [22]. This finding is consistent with previous research conducted by Jones et al. [13] who found that NSLBP episodes lasted less than seven days in the majority of school-children.

The school-children were asked to rate, on average, the perceived intensity of the various episodes of NSLBP they experienced over a 12 months period. The mean intensity was reported to be 4.8 on the VAS. These findings support the conclusions of many epidemiological studies that reported on adolescent LBP being benign or mild [16, 29, 39]. Although with no clear details on the method used to evaluate the intensity of back pain, Ayanniyi et al. [16] observed that 56% (n = 3185) of the school-children with back pain reported the pain to be mild. From a primary prevention perspective, this finding is comforting and partially dismisses the need to lend exaggerated importance to symptoms of NSLBP in adolescents. However, recurrent NSLBP was reported to be intense in females than in males. This is not surprising as males and females have been reported to perceive pain differently [18]. Females are known to have a lower pain tolerance and lower pain thresholds [11].

NSLBP has been reported to occur with or without symptoms of sciatica [40]. The present study found that a small proportion of adolescents (20.9%) had recurrent NSLBP associated with sciatic symptoms. The presence of sciatica in NSLBP patients has been regarded as an important indicator for severe and continuous pain [24, 41]. This suggests that adolescents with recurrent NSLBP characterised by sciatic symptoms deserve attention. In the present study, health-seeking behaviour was associated with sciatic symptoms. Few studies have specifically evaluated the presence of sciatica in adolescents with recurrent NSLBP. In a clinical study conducted on 36 hospitalised patients between the ages of 10 and 18 years, Bockowski et al. [41] found that 52% had NSLBP and sciatica. Despite the small sample size used, the study highlighted the common occurrence of sciatica in adolescents with NSLBP. Watson et al. [30] reported a 31% prevalence of sciatica among 1 446 school-children aged between 11–14 years. Harreby et al. [28] considered sciatica as radiating pain down to the leg to below the knee and reported a prevalence of 4.7% among a cohort of 1 389 Danish school-children aged between 13 and 16 years.

### Critical assessment of the study

The study uniquely investigated lifetime prevalence, point prevalence and prevalence of recurrent NSLBP across all the age categories of adolescents in schools in Zimbabwe. Recurrent NSLBP cases were identified based on a consensus-agreed definition of the condition [21]. However, it is premature to accept and generalise these findings until such a study has been repeated. The response rate from both the school-children and parents was satisfactory. Non-participation bias had no effect on the observed findings. In addition, the study questionnaire had content validity and moderate to substantial reliability.

The main limitation of this study was the cross-sectional and subjective nature of the data collected. A cause-effect relationship cannot be deduced. Reliance on subjective morbidity for retrospective data creates a possibility of recollection bias. This may result in imprecise prevalence figures. However, since pain has been described as a subjective phenomenon, subjective recall has been regarded as the most valid way to assess pain [13]. In addition, two methods were used to assist respondents appreciate the anatomical region of the lower back in an attempt to avoid the influence of recall bias: a direct question and a pre-shaded manikin, with an arrow pointing to a posterior view of the lumber region.

## Conclusion

This study provided evidence that NSLBP is prevalent in the younger age-groups, affecting a number of Zimbabwean adolescents in schools. It is a problem among all the age-groups of adolescents with the prevalence increasing with age. These findings illustrate an untapped health-care problem for the future given that adolescent NSLBP predicts the development of chronic NSLBP in adults. Historically, NSLBP has been reported to be an adult condition that is associated with work-related factors [42, 43]. However, this study has indicated that NSLBP start in early adolescence. The mean age of onset of NSLBP was 14.4 years, peaking earlier in females than in males. The reasons for this are not clear. However, this has been related to puberty-related factors or gender differences in pain reporting. The fact that school-going adolescents are being affected with NSLBP before work-life begins is a disturbing finding that requires further attention. These findings highlight the need for NSLBP awareness or prevention strategies during, or even before the adolescence period. In a small proportion of adolescents, this study showed that NSLBP run a recurrent course, characterised by periods of exacerbations and symptom-free episodes. However, in the majority of adolescents, episodes of recurrent NSLBP are relatively mild and of short duration and unlikely to lead to medical treatment.

## Authors’ information

MC^1^: A lecturer at the University Of Zimbabwe, College Of Health Sciences (UZ-CHS) in the Department of Rehabilitation. MC worked as a clinical physiotherapist at Harare Central Hospital in the Capital City of Zimbabwe, Harare for a period of four years.

NN^2^: A lecturer in the Division of Physiotherapy at the University of Cape Town, South Africa.

## Authors’ original submitted files for images

Below are the links to the authors’ original submitted files for images. Authors’ original file for figure 1 Authors’ original file for figure 2 Authors’ original file for figure 3
