# Supplementary material for: Prevalence and associated characteristics of recurrent non-specific low back pain in Zimbabwean adolescents: a cross-sectional study
Source: BMC Musculoskelet Disord. 2014 Nov 19;15:381. doi: 10.1186/1471-2474-15-381 (PMC4246475; doi:10.1186/1471-2474-15-381)
Supplement: Supplementary file 1 — Authors’ original file for figure 1 [file 12891_2014_2312_MOESM1_ESM.pdf]

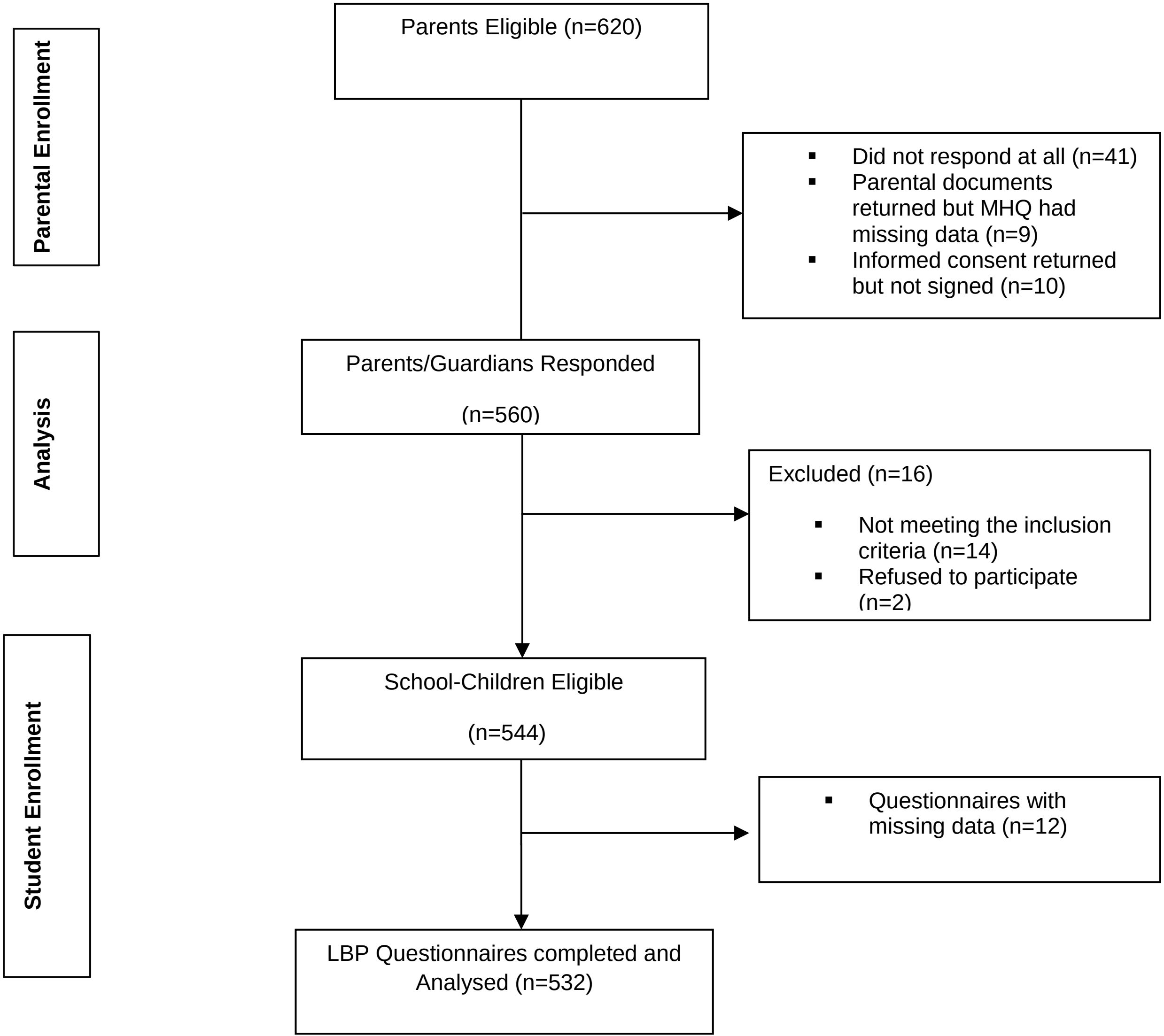
Parental Enrollment
Analysis
Student Enrollment
Parents Eligible (n=620)
Did not respond at all (n=41)
Parental documents returned but MHQ had missing data (n=9)
Informed consent returned but not signed (n=10)
Parents/Guardians Responded
(n=560)
Excluded (n=16)
Not meeting the inclusion criteria (n=14)
Refused to participate (n=2)
School-Children Eligible
(n=544)
Questionnaires with missing data (n=12)
LBP Questionnaires completed and Analysed (n=532)
