# Supplementary figures and images for: Prevalence and associated characteristics of recurrent non-specific low back pain in Zimbabwean adolescents: a cross-sectional study
Source: BMC Musculoskelet Disord. 2014 Nov 19;15:381. doi: 10.1186/1471-2474-15-381 (PMC4246475; doi:10.1186/1471-2474-15-381)

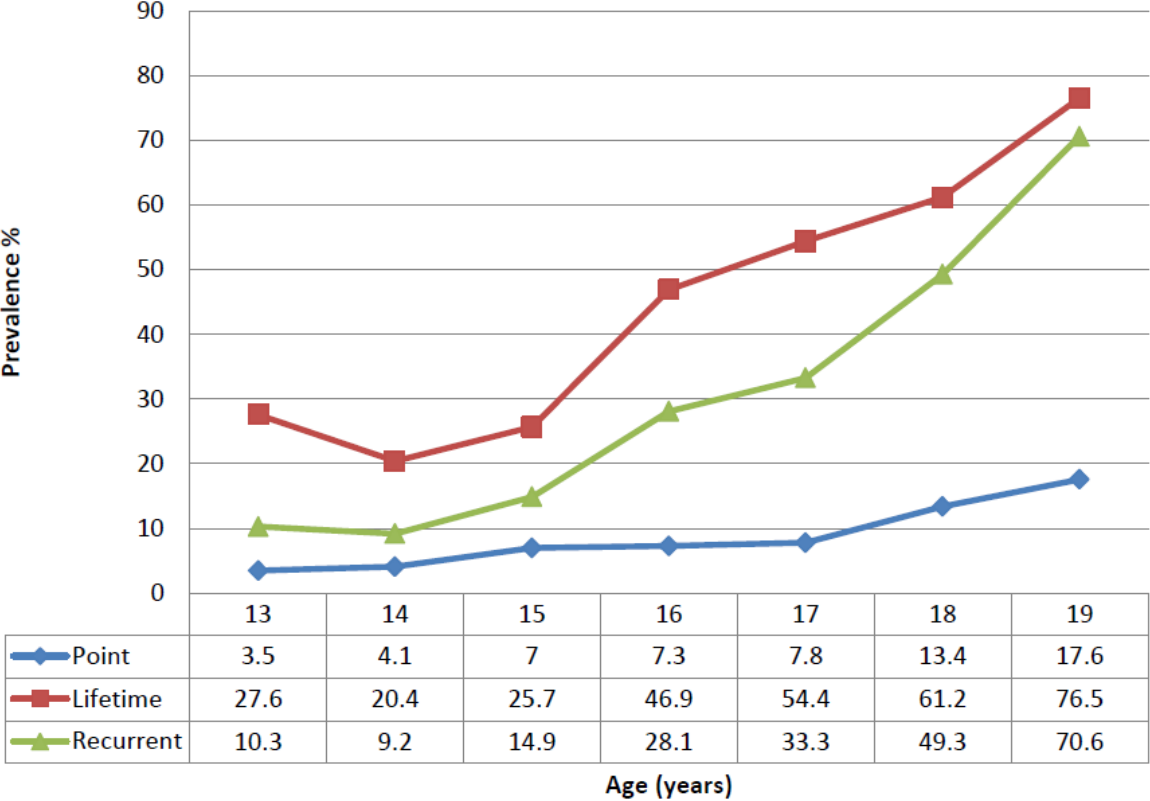

Supplement: Supplementary file 2 — Authors’ original file for figure 2 [file 12891_2014_2312_MOESM2_ESM.pdf]

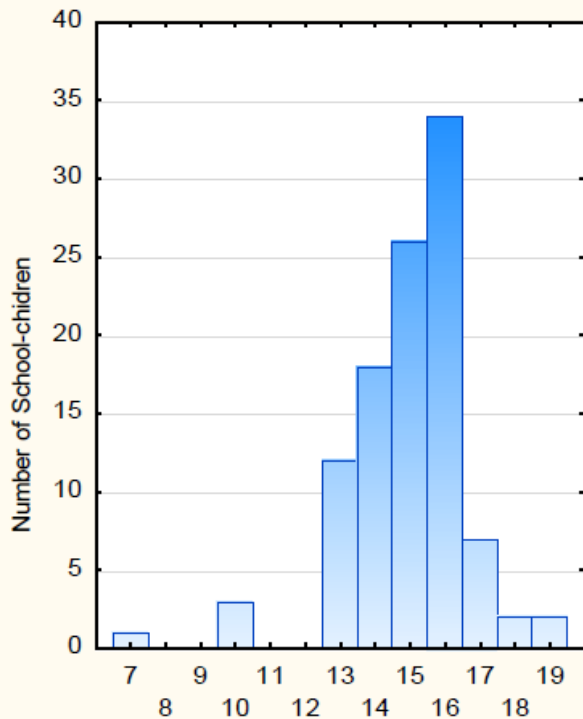

Gender: Male

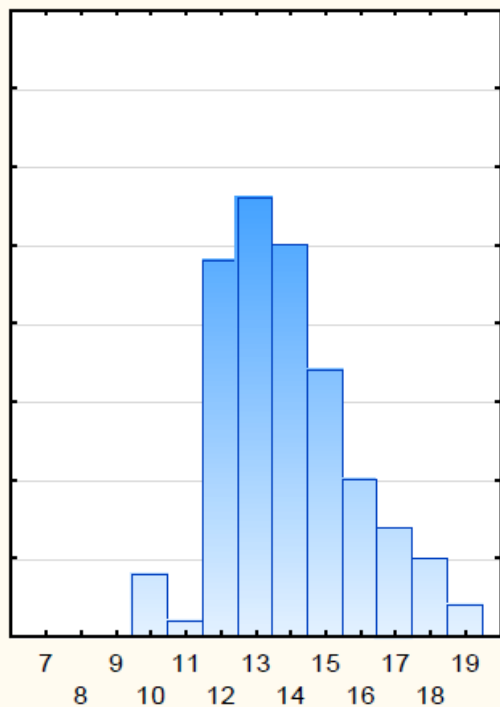

Gender: Female

Age of onset of low back pain

Supplement: Supplementary file 3 — Authors’ original file for figure 3 [file 12891_2014_2312_MOESM3_ESM.pdf]
